# Action Mechanism of Metformin and Its Application in Hematological Malignancy Treatments: A Review

**DOI:** 10.3390/biom13020250

**Published:** 2023-01-29

**Authors:** Yi Zhang, Fang Zhou, Jiaheng Guan, Lukun Zhou, Baoan Chen

**Affiliations:** Department of Hematology and Oncology, Zhongda Hospital, School of Medicine, Southeast University, Nanjing 210009, China

**Keywords:** metformin, leukemia, lymphoma, myeloma, AMPK

## Abstract

Hematologic malignancies (HMs) mainly include acute and chronic leukemia, lymphoma, myeloma and other heterogeneous tumors that seriously threaten human life and health. The common effective treatments are radiotherapy, chemotherapy and hematopoietic stem cell transplantation (HSCT), which have limited options and are prone to tumor recurrence and (or) drug resistance. Metformin is the first-line drug for the treatment of type 2 diabetes (T2DM). Recently, studies identified the potential anti-cancer ability of metformin in both T2DM patients and patients that are non-diabetic. The latest epidemiological and preclinical studies suggested a potential benefit of metformin in the prevention and treatment of patients with HM. The mechanism may involve the activation of the adenosine monophosphate-activated protein kinase (AMPK) signaling pathway by metformin as well as other AMPK-independent pathways to exert anti-cancer properties. In addition, combining current conventional anti-cancer drugs with metformin may improve the efficacy and reduce adverse drug reactions. Therefore, metformin can also be used as an adjuvant therapeutic agent for HM. This paper highlights the anti-hyperglycemic effects and potential anti-cancer effects of metformin, and also compiles the in vitro and clinical trials of metformin as an anti-cancer and chemosensitizing agent for the treatment of HM. The need for future research on the use of metformin in the treatment of HM is indicated.

## 1. Introduction

Hematological malignancies (HMs) mainly include heterogeneous tumors, such as leukemia, lymphoma and myeloma [1,2], and the mechanisms are abnormal hematological cells or immune cells with blocked differentiation and indefinite proliferation followed by biological organismal dysfunction [3]. HMs are different from solid tumors, and due to their specificity, conventional treatment options mainly include chemotherapy, radiotherapy and hematopoietic stem cell transplantation (HSCT) [4]. Although the current conventional first-line treatment has a certain efficacy, primary or secondary drug resistance still often occurs, resulting in a poor overall efficacy with respect to the tumor [5]. Therefore, the search for new therapeutic targets and the development of new treatments remains an important operation with scientific significance in the research of HMs [6].

Metformin is recognized by the World Health Organization (WHO) as an essential drug for the treatment of patients with diabetes and can be widely used in the treatment of type 2 diabetes (T2DM) alone or in combination with other drugs [7]. The most classic action of metformin is to lower hyperglycemia and reduce its clinical symptoms [8]. Due to its low cost and high clinical value in terms of glycemic control and safety, it is now one of the most prescribed drugs in the market [9].

In 1995, Evans et al. first discovered that metformin not only controlled blood glucose in patients with diabetes, but also reduced the risk of malignancies [10]. Since then, various studies in patients with T2DM have shown that metformin use reduces the risk of many cancers [11,12,13,14,15,16], including prostate cancer [17,18], lung cancer [19,20,21], head and neck cancer [22,23], breast cancer [24,25], pancreatic cancer [26], colorectal cancer [26,27,28], endometrial cancer [29], ovarian cancer [30,31,32] and hepatocellular cancer [33,34]. Therefore, this review focuses on the mechanism of action of metformin in cancer prevention and the potential role of this drug in the treatment of patients with HM.

In addition, several studies have supported the therapeutic benefit of metformin in patients with HM. Preclinical data, clinical trial reports, reviews and meta-analyses have reported the effect of metformin as an anti-cancer agent in HM from various perspectives, but these articles reached different conclusions. In this paper, we further focus on the specific mechanisms involved in metformin in cancer treatments, starting with the anti-hyperglycemic effect action mechanism of metformin. Focusing on a collection of published studies on the anti-cancer mechanism of the action of metformin in HM and clinical studies, we affirm the therapeutic potential of metformin for future use in patients with HM.

## 2. Metformin Mechanism of Action

Metformin, a natural derivative of guanidine present in French cloves [35], was first reported to be synthesized by Werner and Bell in 1922 [36,37]. Metformin was first introduced as an anti-hypoglycemic effect agent in the treatment of diabetes by Jean Sterne in 1957 [35], and the following decades saw intense medical interest in the effects of metformin. In 1994, the U.S. Food and Drug Administration (FDA) approved the use of metformin for the treatment of T2DM [38]. In 1998, the UK Prospective Diabetes Study (UKPDS) confirmed the safety and efficacy of metformin as an initial treatment for hyperglycemia in patients with T2DM [39]. This opened an era of using metformin as the first-line treatment for T2DM.

### 2.1. Metformin Anti-Hyperglycemic Effects Mechanism

#### 2.1.1. Systemic Actions of Metformin

Typically, metformin lowers the blood glucose level by inhibiting hepatic glucose production and glucagon signaling, improving insulin sensitivity and increasing peripheral glucose uptake in skeletal muscle, while not affecting glycogenolysis [40,41,42,43,44,45,46,47,48]. These effects normalize chronic hyperglycemia and hyperlipidemia and reduce the damaging effects of metabolic defects on several organs of the body [49,50,51,52].

**Figure 1:**
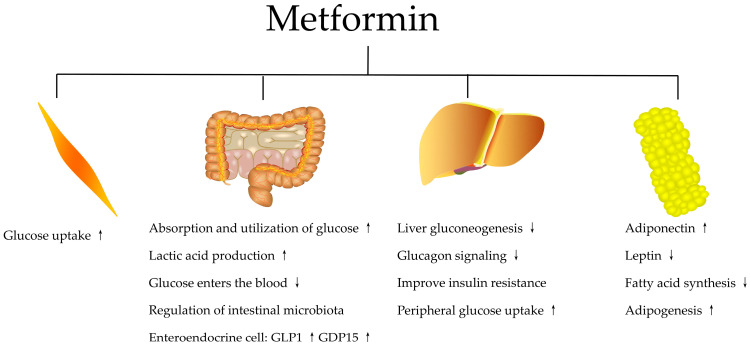
The main systemic effects of metformin to lower blood sugar. GLP1: glucagon-like peptide-1; GDF15: growth differentiation factor 15.

Recent studies have shown that the gastrointestinal tract and its intestinal microorganisms are also involved in the anti-hyperglycemic effects of metformin [53,54]. Metformin increases the intestinal absorption and utilization of glucose in the intestinal cells, which in turn limits the entry of glucose into the bloodstream [55,56]. Altering the composition of the intestinal flora and the secretion of certain key molecules, such as glucagon-like peptide-1 (GLP1) and growth differentiation factor 15 (GDF15), during treatments can affect the gastrointestinal microenvironment and produce beneficial metabolic effects [57,58]. Figure 1 summarizes the mechanisms described above.

#### 2.1.2. Cellular Actions of Metformin

##### AMPK-Dependent Mechanisms

Zhou et al. demonstrated that metformin can activate adenosine monophosphate-activated protein kinase (AMPK) in isolated rat hepatocytes, providing a cellular mechanism for the anti-hyperglycemic effect of metformin [59]. AMPK is an energy sensor that acts in numerous metabolic pathways involved in intracellular energy homeostasis [60].

The activation of AMPK involves many different pathways. In 2000, two independent groups reported that the inhibition of electron transport chain I (ETC I) by metformin decreased nicotinamide adenine dinucleotide (NADH) oxidation and oxygen consumption rates, leading to a decrease in adenosine triphosphate (ATP), an increase in the adenosine monophosphate (AMP)/ATP ratio, and, ultimately, the activation of AMPK [61]. In addition, the inhibition of ETC complex 1 can further inhibit oxidative phosphorylation (OXPHOS), which also causes the activation of AMPK [62]. Shaw et al. demonstrated that metformin can promote AMPK activation by mediating liver kinase B1 (LKB1), the upstream kinase for AMPKα phosphorylation at the amino acid threonine (Thr172), by binding AMPK [63]. In lysosomes, metformin binds to presenilin enhancer-2 (PEN2) and activates AMPK via the axis inhibitor (AXIN)/LKB1–v–ATPase–Ragulator pathway [64,65]. Finally, high intracellular calcium concentrations can promote the activation of calcium/calmodulin-dependent protein kinase II (CaMKII), which leads to AMPK phosphorylation [66,67].

AMPK activation can lead to the inhibition of the mammalian target of rapamycin (mTOR) signaling proteins [68,69]. mTOR is considered to be an important regulatory molecule in controlling the growth and proliferation of diabetes and cancer cells [69,70]. Activated AMPK shuts down ATP depletion, restores cellular homeostasis and converts the cell from an anabolic to catabolic state. At the same time, the metabolic activation of insulin receptor (IR) and insulin receptor substrate 1 (IRS1) increases cellular sensitivity relative to insulin [71]. AMPK phosphorylates acetyl coenzyme A carboxylase (ACC), inhibiting the conversion of acetyl coenzyme A(acetyl-CoA) to malonyl-CoA, leading to reduced hepatic lipogenesis and hepatic steatosis, and improving insulin resistance and hyperglycemia [72,73,74].

##### AMPK-Independent Mechanisms

The same inhibition of gluconeogenesis by metformin has been observed experimentally in mouse hepatocytes lacking the catalytic subunit of AMPK, verifying that metformin can inhibit hepatic gluconeogenesis in an AMPK-independent manner [67].

**Figure 2:**
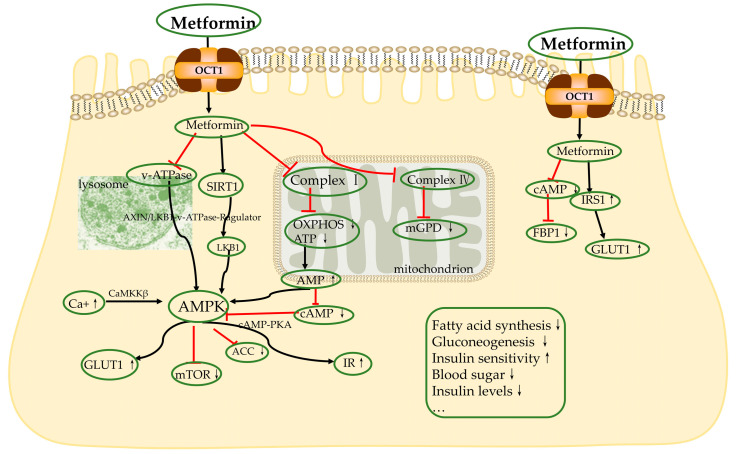
The main cellular actions of metformin to lower blood sugar. OCT1: organic cation trans porter 1; FBP1: fructose-1,6-bisphosphatase; IRS1: insulin receptor substrate 1; GLUT1: glucose transporter protein 1; mGPD: mitochondrial glycerophosphate dehydrogenase; OXPHOS: oxidative phosphorylation; ATP: adenosine triphosphate; AMP: adenosine monophosphate; cAMP: cyclic adenosine monophosphate; IR: insulin receptor; PKA: protein kinase A; ACC: acetyl coenzyme A carboxylase; mTOR: mammalian target of rapamycin; AMPK: adenosine monophosphate-activated protein kinase; CaMKKβ: calcium/calmodulin-dependent protein kinaseβ; LKB1: liver kinase B1; AXIN: axis inhibitor; SIRT1: Sirtuin1; v-ATPase: vacuolar ATP hydrolase.

Some targets can drive metformin effects independently of AMPK, such as ETC I, II and IV; LKB1/AMPK; adenylate cyclase (AC); AMP deaminase; (reduced) nicotinamide adenine dinucleotide phosphate (NADPH) oxidase and mitochondrial glycerophosphate dehydrogenase (mGPD); hexokinase (HK); and voltage-dependent anion channel 1(VDAC1) [75,76,77,78,79,80,81,82,83]. Metformin is able to restrict cytoplasmic access to mitochondrial ATP from these channels, which in turn restricts the cell’s energy supply [84]. For example, metformin can inhibit redox-dependent substrate hepatic gluconeogenesis by blocking ETC IV [85]. Metformin enhances glucose transport to hepatocytes, which is mediated by glucose transporter protein 1 (GLUT1) via the activation of IRS 2, and reduces plasma glucose levels [85]. Metformin similarly promotes Cl^−^ efflux from hepatocytes, depolarizes cell membranes and reduces the uptake of gluconeogenic substrates such as alanine and lactate [75]. Figure 2 summarizes the mechanisms described above.

### 2.2. Metformin Anti-Cancer Mechanism

#### 2.2.1. Activation of LKB1-AMPK-mTOR Signaling Pathway

It is commonly believed that metformin exerts its anti-cancer activity via the LKB1–AMPK–mTOR pathway, and the overexpression of mTOR in this pathway is often associated with the development of numerous diseases such as tumors, among others [86,87,88,89,90]. Metformin activates AMPK after the inhibition of ETC I, inhibits the expression of mTOR and further inhibits the expression of more important factors mediating downstream pro-carcinogenic pathways and the process of tumor development, such as nuclear factor kappa B (NF-κB), interleukin-6 (IL6), mitogen-activated protein kinase (MAPK), Ras and c-MYC [91,92,93]. In animal or in vitro model experiments, high doses (~mM) of metformin inhibited mitochondrial respiratory chain complex I, leading to an increase in the AMP/ATP ratio and prompting the phosphorylation and activation of the upstream kinase LKB1, while low doses of metformin (~μM) activated AMPK via the lysosomal pathway [65]. The mammalian target of rapamycin complex 1 (mTORC1) activation by metformin in the lysosomal pathway via the Ras-associated guanosine triphosphatase (GTPase) completes the regulation of AMPK, which is independent of the AMP/ATP ratio [65,94,95,96]. The activation of the LKB1–AMPK–mTOR signaling pathway can usually mediate the following cellular responses.

1. Regulation of epigenetic modifying enzyme activity [97]: AMPK directly and indirectly regulates histone acetylation and alters gene expression patterns via the epigenetic regulation of various chromatin functions [98,99]. Activated AMPK phosphorylates a variety of substrates including histone acetyltransferases (HATs), histone deacetylases (HDACs) and deoxyribonucleic acid (DNA) methyltransferases (DNMTs), often leading to their inhibition [98,99,100];
2. Activation of p53: Activated P53 promotes apoptosis, cycle arrest, autophagy and the inhibition of the phosphoinositide 3-kinase (PI3K)–protein kinase B (AKT) signaling pathway [101,102]. The effect of P53 in tumor apoptosis was also associated with the reduced expression of transcription factor specificity protein 1 (Sp1), Sp3, Sp4 and oncogenes (B-cell lymphoma-2 (BCL-2), mTOR, vascular endothelial growth factor (VEGF) and MYC, etc.) [103,104];
3. Activation or inhibition of the expression of other cancer-related signaling pathways:
  3.1 Activation of the AMPK-forkhead box O1/3 (FOXO1/3) signaling pathway [105,106,107]: FOXO is a proliferation-associated transcription factor, and the activation of FOXO3a has been shown to be required for the pro-apoptotic and chemotherapeutic effects of metformin in a variety of tumor models [108,109];
  3.2 Suppression of Hippo pathway expression [110,111]: In some (cancer) models, metformin has been shown to repress the activity or expression of yes-associated protein (YAP)/tea domain transcription factor (TAZ), which are two central effectors of the Hippo pathway that are mediated by AMPK [112,113,114,115,116,117];
  3.3 Inhibition of the receptor tyrosine kinase (TK) pathway: this includes epidermal growth factor receptor (EGFR) signaling, further targeting downstream effectors AKT, mTOR, extracellular regulated protein kinases (ERK), etc. [118];
  3.4 Inhibition of the PI3K/AKT pathway: in vitro and in vivo experiments showed that the PI3K/AKT signaling pathway inhibited by metformin stimulated the expression of phosphatase and tensin homolog (PTEN) and inositol triphosphate 3 (IP3) [119];
  3.5 Affecting natural killer (NK) cell signaling pathways: the anti-cancer properties of specific immune regulation, the inhibition of cell proliferation and the induction of cell cycle arrest are exerted via the inhibition of tumor cell metastasis, endothelial cell proliferation and the alteration of NK cells’ ligand expression on the surface of tumor cells [120,121,122,123];
  3.6 Stimulation of the Sirtuin1 (SIRT1) signaling pathway: the upregulation of SIRT1 by AMPK can lead to the amelioration of oxidative stress and a reduction in DNA damage [124,125];
4. Inhibition of cancer stem cells (CSCs): CSCs are cancer cells with unlimited renewal capacity. A number of studies have shown that metformin inhibits the biological activity of CSCs in a variety of tumors, including gastric, endometrial and ovarian cancers [126]. Subsequently, metformin activates additional signaling pathways for targeting CSCs via the activation of AMPK, which include PI3K–AKT–mTOR, insulin-insulin growth factor1 (IGF1), MAPK, sonic hedgehog (Shh), Wnt, TGFB, Notch, Hippo and NFkB pathways [126];
5. Reducing IRS1 phosphorylation [127]: activated AMPK inhibits hyperinsulinemia-associated tumor activity by reducing the circulating insulin levels and targeting the insulin–IGF1–PI3K signaling axis [127,128];
6. Mitigation of hypoxia and other tumor responses caused by hypoxia: Metformin leads to slow tumor growth by decreasing the expression of hypoxia-inducible factor-1α (HIF-1α), which in turn decreases the expression of HIF1-targeted genes [129,130,131,132]. The inhibition of HIF-1α simultaneously suppresses the immunosuppressive activity of myeloid-derived suppressor cells (MDSCs) and improves T-cell immunity in the tumor microenvironment (TME) [133,134,135]. These immune responses are ultimately also involved in the inhibition of angiogenesis [136];
7. Inhibition of adipogenesis: The cancer cells themselves require more nutrients and energy; thus, the rate of adipogenesis is higher [137]. AMPK inhibits lipogenesis by targeting the activity or expression of many lipogenic enzymes. On the one hand, AMPK phosphorylates and inhibits 3-hydroxy-3-methylglutaryl CoA reductase (HMGCR), which catalyzes the rate-limiting step in cholesterol synthesis. On the other hand, AMPK phosphorylates and inactivates acetyl CoA carboxylase (ACC), the main enzyme involved in the biosynthesis of fatty acid and HMGCR, resulting in the inhibition of cholesterol biosynthesis [138]. In addition, AMPK also phosphorylates sterol regulatory element binding protein-1c (SREBP-1c) at Ser-372, which restricts its cleavage and nuclear translocation. This process results in the downregulation of SREBP-1c target genes, including those encoding ACC1 and fatty acid synthase (FASN), and leads to reduced lipogenesis [139];
8. Activation of ataxia telangiectasia-mutated gene (ATM), which leads to the activation of DNA damage repair pathways and the inhibition of tumor growth [140].

#### 2.2.2. Activation of AMPK-Independent Signaling Pathways

Some of the pathways controlled by metformin can also be modified in a manner that is not dependent on AMPK activation. Metformin can promote crosstalk between apoptosis and autophagy in multiple ways to accomplish anti-tumor effects [141]. These include downregulating the signal transducer and the activator of transcription 3 (STAT3)/Bcl2/Beclin1 signaling pathway activity in cancer cells, disrupting unfolded protein response (UPR) transcription, blocking the oncogenic Wnt signaling pathway, regulating caspase enzyme activity, increasing reactive oxygen species (ROS) production by cancer cells, inhibiting the activation of mTORC1 by Rag GTPases and inhibiting glutaminase activity [142,143,144,145,146,147,148,149]. Shen et al. found the preferential inhibition of cell-cycle-related proteins among the broad inhibitory effects of metformin on protein synthesis [150]. Specifically, the metformin activation of the p53-regulated in the development DNA damage response 1 (REDD1) axis caused an independent inhibition of AMPK by mTOR, leading to a decrease in cell-cycle protein D1 (cyclin D1) expression, which in turn inhibited the G0/G1 cell cycle [66,151]. In addition, metformin induces the expression of dicer, an enzyme that processes microRNAs (miRNAs) precursors into mature miRNAs, as a means of regulating miRNAs to exert anti-cancer effects [152]. Numerous experiments have shown that metformin inhibits tumorigenesis and tumor growth by altering the expression of specific miRNAs in human breast, pancreatic, prostate, renal cell, lung, hepatocellular, bile duct, small intestine and human oral cancers [153,154,155,156,157,158,159,160,161,162]. The repression of different miRNAs can directly or indirectly induce apoptosis, cycle arrest and other phenomena. Metformin reduces stressor-induced ROS production, activates endogenous repair systems, prevents ROS toxicity, protects cells and ameliorates genomic instability and possible cancer risk [163]. Metformin reduces the expression of the P-glycoprotein (P-gp) encoded by multidrug resistance1 (MDR1), and, via this action, it blocks drug efflux from cancer cells, allowing metformin to be used as an adjuvant relative to classical chemotherapy [164].

#### 2.2.3. Altered Energy Metabolism of Tumors

Cancer cells have a different pathway for energy production. Even under aerobic conditions, the tricarboxylic acid cycle (TCA cycle) and OXPHOS are restricted, and they favor lactic acid fermentation, called the “Warburg effect” [165,166]. Many studies observed that mitochondrial OXPHOS is the main source of ATP in most cancer tissues [167]. Metformin can reduce the availability of glucose to cancer cells by causing a metabolic shift from OXPHOS to glycolysis via the inhibition of mitochondrial function [61]. Metformin targets mitochondrial glycerol 3-phosphate dehydrogenase (mGPDH), causing the inhibition of mitochondrial function while impairing the production of dihydroxyacetone phosphate (DHAP), which can reduce the process of glucose production from glycerol [80]. In addition, metformin targets mitochondrial integrity by modulating calcium flux in cancer cells [168]. Inducing endoplasmic reticulum stress and releasing calcium into the cytoplasm of cells induces higher calcium uptake by mitochondria, ultimately leading to mitochondrial swelling.

#### 2.2.4. Improved Immune and Inflammatory Response

Inflammatory cells are essential players in the tumor process, promoting the proliferation, survival and migration of tumor cells [169]. In addition to targeting cancer cells, metformin has been shown to target immune cells in the tumor microenvironment, such as CD8^+^ T cell, regulatory cells (Tregs), MDSCs and tumor-associated macrophages (TAMs), which may contribute to the expression of anti-tumor activities [170].

The NF-κB signaling pathway is a key regulator of immunity and plays an important role in tumor progression. Metformin inhibits the NF-κB signaling pathway and reduces the levels of inflammatory cytokines including tumor necrosis factor-α (TNF-α) and IL-6 [171]. In addition, metformin inhibits ETC I, further inhibiting ROS and interleukin-1β (IL-1β) production via lipopolysaccharide (LPS)-activated macrophages [172]. Metformin can directly and indirectly alter the function of CD8^+^ T cells, blocking T cell depletion by increasing the number of CD8^+^ T lymphocytes (CD8^+^ TILs) and protecting them from apoptosis and exhaustion [173,174]. In addition, metformin can reduce programmed cell death protein 1 ligand 1 (PD-L1) in tumor cells via multiple pathways, leading to enhanced cytotoxic T lymphocyte (CTL) activity [175]. In addition, metformin helps the body produce sustained anti-tumor immunity by acting on multiple immune pathways.

#### 2.2.5. Reduced Tumor Vascular Metastasis and Invasion

**Figure 3:**
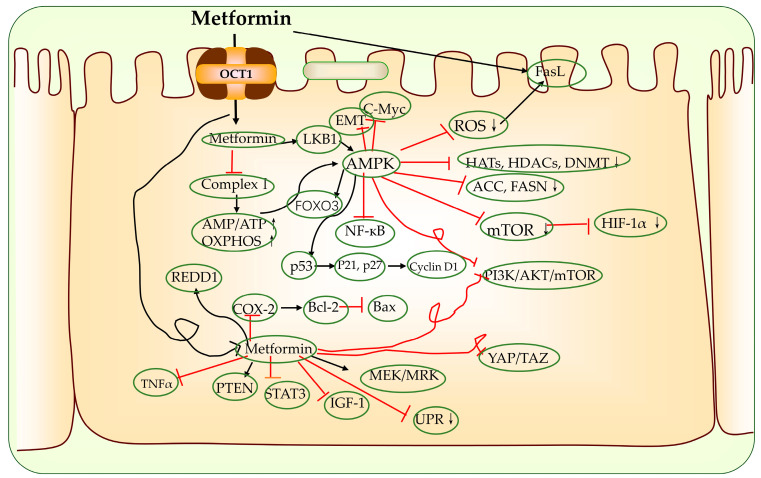
The main cellular anti-cancer actions of metformin. OCT1: organic cation transporter 1; AMP: adenosine monophosphate; ATP: adenosine triphosphate; OXPHOS: oxidative phosphorylation; REDD1: deoxyribonucleic acid (DNA) damage response 1; TNFα: tumor necrosis factor α; PTEN: phosphatase and tensin homolog; STAT3: transcription 3; IGF-1: insulin growth factor-1; YAP: yes-associated protein; TAZ: tea domain transcription factor; PI3K: phosphoinositide 3-kinase; AKT: protein kinase B; mTOR: mammalian target of rapamycin; COX-2: cyclooxygenase-2; Bcl-2: B-cell lymphoma-2; Bax: Bcl-2 associated x; NF-κB: nuclear factor kappa B; AMPK: adenosine monophosphate-activated protein kinase; ROS: reactive oxygen species; LKB1: liver kinase B1; FOXO3: forkhead box O3; HATs: histone acetyl-transferases; HDACs: histone deacetylases; DNMT: deoxyribonucleic acid (DNA) methyltransferase; ACC: acetyl coenzyme A carboxylase; HIF-1α: hypoxia-inducible factor-1α; Cyclin D1: cell cycle protein D1; EMT: mesenchymal transition; FASN: fatty acid synthase; UPR: unfolded protein response; MEK: mitogen-activated protein kinase; MRK: extracellular-related kinase; FasL: Fas ligand.

Several studies investigated the mechanism by which metformin inhibits cancer invasion and metastasis by inhibiting the EMT signaling pathway [176]. Metformin inhibits the epithelial to mesenchymal transition (EMT) signaling pathway via multiple pathways, including blocking cyclooxygenase-2 (COX-2)-mediated prostaglandin E2 (PGE2) production, reducing Snail protein expression, inhibiting B-cell-specific Moloney murine leukemia virus integration region 1 (Bmi-1, also known as RNF51 or PCGF4) and reducing IL-6 [177,178,179,180,181,182]. In addition, metformin inhibits angiogenesis and attenuates tumor migration by attenuating angiogenic stimulation, downregulating VEGF expression, inhibiting HIF-1α-induced angiogenesis-related factor expression and reducing the signaling of platelet-derived growth factor B (PDGF-B) and PDGF-receptorβ (PDGF-Rβ) [183,184,185,186]. Figure 3 summarizes the mechanisms described above.

## 3. Therapeutic Application of Metformin in Hematological Malignancies

A large number of different types of studies have shown that metformin is a potential therapeutic target for patients with leukemia, lymphoma and multiple myeloma, and it is expected that the pleiotropic effects of this drug could act on multiple targets [53,187,188,189,190,191,192,193,194]. Herein, we discuss experimental ex vivo studies and clinical observations of metformin in different hematologic malignancies. We compiled tables of preclinical applications of metformin in malignant hematological during the last decade, divided into in vivo animal experiments (Table 1), in vitro cellular experiments in leukemia (Table 2), lymphoma (Table 3) and myeloma (Table 4).

### 3.1. Leukemia

#### 3.1.1. Acute Myeloid Leukemia (AML)

AML is the most common acute leukemia in adults and is characterized by the uncontrolled proliferation of leukemic cells that inhibit normal bone marrow hematopoiesis following varying degrees of differentiation blockage with respect to bone marrow progenitor cells [239].

Since metformin was shown to interfere with the proliferation and clonal activity of AML cells without affecting normal CD34^+^ HSCs, numerous experiments have begun to further explore the anti-AML effects of metformin [240]. Metformin accelerates AML cell apoptosis by inhibiting OXPHOS, decreasing FOXM1, activating the LKB1–AMPK signaling pathway and dephosphorylating eukaryotic initiation factor 4E binding protein 1 (4E-BP1) [213,224,241,242]. In acute progranulocytic leukemia (APL) cell lines, metformin induced c-Myc degradation and apoptosis via the MEK–ERK signaling pathway [243]. In addition, metformin combined with sorafenib, cytarabine and venetoclax showed synergistic enhancement effects in AML treatment, and these effects were demonstrated by mTOR inhibition and the regulation of apoptosis-related proteins [196,199,221]. Clinically, a retrospective hospital cohort study found that although metformin users did not perform better than non-users in overall survival (OS) and disease-free status, they performed better than insulin and oral diabetes medication-treated AML patients [244].

#### 3.1.2. Chronic Myeloid Leukemia (CML)

CML is characterized by the presence of the Philadelphia (Ph) chromosome with a reciprocal translocation between chromosomes 9 and 22, and this translocation generates the BCR/ABL fusion gene [245]. A tyrosine kinase is produced by the BCR/ABL fusion gene, which drives tumorigenesis and prevents apoptosis by activating downstream signals [246].

After AMPK activation, metformin inhibited the proliferation and clonal activity of different CML cell lines, including the T315I BCR-ABL mutant CML line expressing imatinib resistance [247]. With regard to drug combinations, experiments have shown that the combination of nilotinib and metformin is more effective than the combination of nilotinib and c-Jun N-terminal kinase (JNK) inhibitors [208].

An observational study of patients with newly diagnosed chronic-phase CML showed that the proportion of patients reaching complete cytogenetic response (CCyR) was 100% and 73.6% in the metformin and non-metformin groups, respectively, with similar numbers reaching major molecular response (MMR) and complete molecular response (CMR) between the two groups. The median time for achieving MMR and CMR was shorter in the metformin group compared to the non-metformin group (11.1 months vs. 19.5 months; 37.4 months vs. NR), indicating that metformin used together with TKI therapy may increase the proportion of CML patients achieving CCyR and shorten the time to MMR and CMR [248].

#### 3.1.3. Acute Lymphocytic Leukemia (ALL)

ALL is aggressive, with the clonal proliferation of immature lymphocytes at different stages of differentiation that, if not controlled, can cause a destructive infiltration of the bone marrow [249]. In adults, 75% of cases develop from the precursors of the B-cell lineage and the rest consist of malignant T-cell precursors [250].

Metformin increases the chemosensitivity of ALL by inhibiting the AKT–mTOR pathway via the activation of AMPK [251,252]. In T-ALL cells, metformin-stimulated AMPK inhibited mTOR to trigger cellular autophagic responses by a mechanism involving miRNA-19 overexpression [225,253]. Metformin-triggered apoptotic effects also involve the AMPK-dependent activation of the endoplasmic reticulum (ER) stress/UPR cell death pathway and the apoptotic mediators including inositol-requiring enzyme1 (IRE1) and C/EBP-homologous protein (CHOP) [225]. In addition to AMPK, the expression of other important metabolic kinases is also regulated by metformin in ALL cells. Metformin regulates the expression of protein kinase C-ε(PKCε) and δ in responding cells, leading to the downregulation of PKCδ and control of PKCε [254]. The altered balance between PKCε and PKCδ has important implications for tumor cell survival, as both kinases control the nuclear factor erythroid 2-related factor (Nrf2) transcription factor [255]. In adult T-cell leukemia (ATL), metformin activates LKB1–AMPK and inhibits leukemic proliferation by reducing Tax expression [256]. Leukemic stem cells (LSCs) are rare cells with leukemic origins that have an intrinsic resistance mechanism relative to chemotherapy [257]. In T-ALL cells, metformin can target the Hoescht 33342^low^ side population and the CD34^+^CD7^−^CD4^−^ subpopulation, which are rich in LSCs [251].

In ALL, metformin can be used in combination with anthracyclines (doxorubicin and adriamycin), dexamethasone, imatinib, proviral integration sites for Moloney murine leukemia virus (PIM)-1/2 kinase inhibitors, thiazolidinediones and other drugs to exert synergistic effects, reducing tumor cell growth and survival by stimulating various signaling pathways and showing a cumulative effect [193,203,222,225,251,252]. The use of metformin in ALL treatment can help reduce the dose of adriamycin that is necessary to prolong remission and reduce the cardiotoxicity of anthracyclines [258]. There are, of course, different phenomena. Studies have shown that the combination of metformin and cisplatin exerts an antagonistic effect in ALL, suggesting that metformin and oncology drugs are not synergistic in one way or the other [206].

In a prospective study of 102 patients with new-onset Ph^−^ B-cell leukemia (NCT03118128), metformin combined with chemotherapy was shown to be effective in ALL patients with elevated ABCB1 gene expression levels (*p* = 0.025), and in the metformin user group the drug was protective against treatment failure (odds ratio (OR) 0.07, 95% confidence interval (CI) 0.0037–1.53) and early relapse (OR 0.05, 95% CI 0.0028–1.153) in the metformin user group [259]. A phase 1 study (2018) exploring metformin in combination with induction chemotherapy for relapsed/refractory (R/R) ALL showed that the combination of metformin and vincristine, dexamethasone, PEG-asparaginase and doxorubicin (VXLD) was tolerable in the 14 included patients; the overall treatment induced ER stress; and toxic reactions occurred at all dose levels of metformin [260]. In the randomized controlled trial of an intensive insulin regimen in patients with hyperglycemic ALL (NCT00500240), the trial was terminated early, although this ALL clinical outcome was invalid. However, a secondary analysis showed that the use of metformin and/or thiazolidinedione was associated with increased progression-free survival (PFS) compared to other drug combinations and may improve the patient prognosis [261]. In a small phase I clinical trial in patients with ALL, the addition of metformin to standard chemotherapy was well tolerated. The unselected use of metformin during this treatment was the only variable considered to have a significant adverse prognosis (*p* = 0.55). The Cox regression showed that the addition of metformin reduced the risk of recurrence by 56% [262]. A retrospective analysis reported on the safety of the metformin treatment for hyperglycemia control in 17 patients with ALL during the induction period. The data showed no significant toxicity of metformin in all patients, with only one patient failing to control glycemia, suggesting that metformin can be used in clinical ALL patients for glycemic control [263].

#### 3.1.4. Chronic Lymphocytic Leukemia (CLL)

CLL is a common leukemia in western countries, and metformin has been found to be cytotoxic to both resting and activated CLL cells [264]. The cytostatic effect of metformin on activated CLL cells was manifested by the reduced expression of proliferation and proliferation-related molecules. In these cells, metformin inhibited the activation-induced upregulation of chemokine receptors and adhesion molecules that can synergistically stimulate mitogenic activity and cell homing in lymphoid tissues [265]. Metformin inhibits the activation of transcription factors in the CLL pro-survival and pro-activation pathways by activating AMPK and reducing glucose metabolism, further impeding CLL cell survival, activation and clonal expansion [265,266,267,268,269,270]. CLL cells are more dependent on the protective activity of BCL-2 anti-apoptotic protein than normal cells. This also explains the higher sensitivity of CLL cells to metformin than normal lymphocytes [271]. Metformin modulates the glycolytic capacity of CLL cells and limits the availability of extracellular glucose, thereby increasing the sensitivity of CLL cells relative to the cytostatic effects of metformin [264]. The combination of classical anti-CLL drugs fludarabine, ritonavir and ABT-737 with metformin may enhance its cytotoxicity by altering mitochondrial activities and other mechanisms of action, and can exert synergistic effects [53,272].

### 3.2. Multiple Myeloma (MM)

MM is a malignant plasma cell proliferative disease with major symptoms including elevated blood calcium levels, renal insufficiency, anemia and bone damage (CRAB) [273]. In vitro and in vivo experiments demonstrated a direct anti-tumor effect of metformin on myeloma [92,191,194,201,202,235,237,274].

Metformin inhibits the proliferation of myeloma cells by stimulating apoptosis and cell-cycle arrest by a mechanism involving the dual inhibition of the AMPK activation-mediated mTORC1 and mTORC2 pathways [201]. In terms of cell proliferation, metformin inhibits the expression of a range of signaling pathways such as IGF1, PI3K, AKT and downstream mammalian rapamycin [190]. Metformin reduces insulin and IGF1 production, increases insulin sensitivity and is thought to reduce the progression of monoclonal gammopathy of undetermined significance (MGUS) relative to multiple myeloma [275,276]. Metformin specifically reduced IL-6R expression via AMPK, is mediated by mTOR and showed synergistic effects with common anti-myeloma drugs [92]. Hypoxia stimulates the migration and homing of malignant plasma cells relative to new bone marrow niches, promoting osteolytic bone destruction and MM-induced angiogenesis [277,278,279,280]. Metformin prevents HIF-1α accumulation in hypoxia and slows disease progression by inhibiting the activity of ETC I [131,281,282]. In addition, cellular experiments demonstrated that metformin could exert anti-MM cellular effects by downregulating the STAT3 signaling pathway; activating the ERK1/2, MAPK and NF-κB pathways; blocking glucose-regulated protein 78 (GRP78) promoter reporter activation; inhibiting UPR activation; and so on [194,238,283].

The combination of metformin and dexamethasone synergistically eliminates MM cells by reducing AKT–mTOR signaling and inhibits cell proliferation [191]. In animal models, metformin significantly synergized with ritonavir to reduce the tumor burden of multiple myeloma [53]. Concomitant treatments with metformin and bortezomib inhibit the effect of UPR on GRP78, impair autophagosome formation and increase apoptosis [194].

Several studies have shown that being overweight, having high insulin levels and a history of diabetes are poor prognostic indicators for patients with multiple myeloma [284]. A retrospective study of 1240 patients with MM evaluated the impact of diabetes mellitus (DM) and antidiabetic drugs on clinical outcomes in MM, and the data suggested that metformin is associated with reduced death in progressive MM and that it improves MM-related prognosis [192]. In a retrospective study of 3287 U.S. veterans with diabetes diagnosed with MGUS, metformin users demonstrated the potential for a lower risk of progression relative to multiple myeloma than non-users (hazard ratio (HR) 0.47, 95%CI 0.25–0.87) [189]. These findings were extended by a case control study by Boursi et al. Using information from a U.K. database, Boursi et al. found that antidiabetic drugs may be associated with a reduced risk of active MM in patients with MGUS diabetes and with a reduced but not significant risk of MM among patients with diabetes exposed to metformin (OR 0.39, 95%CI 0.14–1.13), with the effect being observed mainly in patients who had been on treatment for at least 2 years, rather than those who had been on treatment for a shorter period of time [190]. Of course, there are studies that have come to different conclusions. One study of 2363 MGUS patients and 9193 controls showed no significant reductions in the risk of MGUS in patients with diabetes treated with metformin alone compared to those without diabetes (OR 0.77, 95%CI 0.56–1.05) [285].

### 3.3. Lymphoma

The incidence of lymphoma is currently increasing year by year [286]. In patients who have relapsed or are resistant to conventional chemotherapy, especially those with an aggressive subtype, the prognosis remains suboptimal. Shi et al. provided evidence of the ex vivo activity of metformin acting on human lymphoma cells, demonstrating that the metformin activation of AMPK inhibits the growth of B- and T-cell lymphomas via the inhibition of mTOR signaling, suggesting that metformin can be used in the treatment of lymphoma [193]. At the same time, metformin can enhance the tumor suppressive effect of common drugs for treatments such as asdriamycin, dacarbazine, venetoclax and rituximab [200,230,287].

Diffuse large B-cell lymphoma (DLBCL) is the most common histologic subtype of non-Hodgkin’s lymphoma (NHL) [288]. The mechanisms by which metformin affects DLBCL cell proliferation are multifactorial. A portion of DLBCL survival and progression is highly dependent on B-cell receptor (BCR) signaling [289,290,291]. Metformin blocks cholesterol synthesis by inhibiting the key cholesterol synthesis-related enzyme hydroxy-3-methylglutaryl coenzyme A synthetase 1 (HMGCS1), which ultimately leads to the intracellular inhibition of cholesterol-dependent BCR and its downstream signaling, resulting in a blockage of BCR signaling [292]. Metformin acts on lymphoma cells to produce an increase in reactive oxygen species, and it promotes the loss of mitochondrial membrane permeability [200]. Metformin induced a G1 cell-cycle block and showed a decrease in cellular proteins C-MYC, cyclin dependent kinase 2 (CDK2), E2F and proliferating cell nuclear antigen (PCNA) [200]. Burkitt’s lymphoma (BL) is a rare and aggressive subtype of NHL [293]. Metformin was found to strongly affect the viability of DAUDI cells (a human BL cell line), and peripheral blood lymphocytes (PBL) were unaffected by treatments, suggesting that metformin has the ability to selectively target cancer cells [228]. BL exhibits a specific metabolic shift towards aerobic glycolysis, leading to a strong change in metformin that can kill BL cells by determining glucose use [228].

The results of clinical studies on the role of metformin in lymphoma are mixed. A study by Wang et al. found no evidence of significant improvements in event-free (EFS), lymphoma-specific (LSS) and OS-associated metformin use in terms of prognostic improvement in newly diagnosed DLBCL and follicular lymphoma (FL) [294]. In addition, two large retrospective studies and one case–control study involving the action of metformin in patients with lymphoma showed similar results in that metformin exposure as a pre-chemotherapy agent had no significant effects on lymphoma survival [188,295,296].

There are other studies that disagreed with the above observations. A retrospective study on metformin action in patients with DLBCL in T2DM showed that the use of metformin did help significantly improve CR rates and ORR, in addition to PFS. In addition, the metformin group showed a trend toward improved OS, although the difference did not reach a statistically significant level [292]. A retrospective analysis of DLBCL patients receiving first-line chemotherapy showed that the use of metformin improved the PFS from 60 to 90 months and OS from 71 to 100 months in patients with diabetes compared with those not using metformin [200]. In a 10-year study in Taiwan on whether the use of metformin affects the risk of NHL, the risk of NHL was significantly lower in patients with T2DM treated with metformin-based antidiabetic drugs compared with non-metformin antidiabetic drugs [297]. One team also found significantly higher overall long-term survival for lymphoma in the metformin group (5.89 vs. 1.29 years, *p* < 0.001) than in the non-metformin group. Metformin was associated with a significant positive impact in improving overall survival in patients with type 2 diabetes and lymphoma [298]. In patients with DLBCL with DM, metformin improved the PFS (94 vs. 55.4 mon, *p* = 0.007) and OS (100 vs. 70.5 mon, *p* = 0.031) compared with other anti-diabetic agents [299]. A prospective study by Dr. Zhao’s group also found that the use of metformin prolonged survival in DLBCL [300].

### 3.4. Ongoing Studies

According to ClinicalTrials.gov, there are currently 17 trials of metformin and HM. Metformin is being investigated in HM mainly as an adjuvant therapy. Five of these seventeen studies have been completed. These studies are shown specifically in Table 5.

## 4. Adverse Effects of Metformin

Metformin can cause various adverse reactions, which cannot be ignored in the treatment process. Digestive disturbances (nausea, vomiting, diarrhea, stomach upset, etc.) are the most common side effects of metformin and, while severe symptoms may lead to discontinuation in 5–10% of metformin users, these side effects are usually transient and mild [301,302,303]. This can be easily resolved by gradually titrating the dose, taking the drug with food or switching to an extended-release formulation [304]. The effects of metformin on gastrointestinal function may lead to vitamin B12 deficiency [305]. A cross-sectional study found a significant correlation between metformin ≥ 1500 mg/d and vitamin B12 deficiency [306]. This effect was more common in patients who had been on high doses for three years [307]. It can be reversed by discontinuation or calcium supplementation [308].

The most serious adverse effect is lactic acidosis, but this complication is very rare, with an incidence of 1/30,000, and it is mainly seen in patients with diabetes with renal and hepatic abnormalities [309]. Metformin increases plasma lactic acid levels by inhibiting mitochondrial respiration in the liver and inhibiting the conversion of lactic acid into glucose in the liver [310,311]. Therefore, excessive metformin can lead to a severe hypoglycemia. The true role of metformin in the development of metformin-associated lactic acidosis (MALA) in the clinical setting is unclear. Some trials have shown that the incidence of lactic acidosis in metformin users is not significantly different from that in patients treated with other oral anti-hyperglycemic effects agents [312]. The risk factors for MALA include impaired renal function resulting in poor metformin clearance, impaired liver function resulting in poor lactate clearance and increased lactate production, such as sepsis [313]. Overall, mortality in patients taking metformin or even developing lactic acidosis appears to be related to the underlying disease rather than metformin being a toxic drug [314]. As metformin is excreted by the kidneys, chronic kidney disease is considered a major risk factor for lactic acidosis [75,315]. There is insufficient evidence to support the safe use of metformin in people with eGFR values below 30 mL/min/1.73 m^2^. Therefore, it is generally recommended to discontinue metformin if renal function is below this level [316].

In-depth studies have identified several unexpected risks associated with metformin, such as birth defects in offspring, cognitive deficits in rodents, acute hepatitis, cholestasis and hemolytic anemia [317,318,319]. These require further confirmation of adverse effect results and revision of clinical guidelines for precisely defined indications and doses of metformin. A fact sheet issued to the public by the UK Teratology Information Service states that there are no known risks associated with the use of metformin during pregnancy [320]. However, there is limited evidence that metformin can affect anthropometric parameters in offspring with unknown long-term consequences. Therefore, the risks and benefits of continuing metformin during pregnancy should be carefully evaluated and individualized [321]. Metformin therapy appears to be safe for most patients in the early stages of COVID-19 infection. However, immediate discontinuation of metformin as soon as COVID-19 infection becomes severe is a reasonable and practical preventive measure for metformin. This approach may reduce the likelihood of life-threatening complications from COVID-19 or lactic acidosis events associated with metformin [322].

## 5. Limitations of Metformin in Cancer Treatment

Although some studies support the effectiveness and feasibility of metformin for the treatment of cancer, metformin is primarily used in patients with diabetes. In clinical use, the feasibility of metformin alone or in combination with other cancer treatment regimens may be limited to patients with non-diabetes because it may result in adverse effects that are intolerable to some patients [323]. For example, patients receiving both metformin and radiation therapy experienced increased local toxicity, increased frequency of treatment interruptions and desquamation or dermatitis [324,325,326]. The report also identified time-related biases in observational studies that tended to exaggerate metformin’s anti-tumor effects [327,328]. The anti-tumor effects of metformin increased with increasing doses, as compiled from experimental data. Many experiments have used metformin at concentrations much higher than the conventional dose for diabetes treatment [329]. These high doses are not suitable for actual clinical use due to potential drug toxicity. At the same time, the efficacy of metformin as an anti-cancer drug is highly dependent on the glucose concentration in the tumor microenvironment [330,331].

In addition, many factors influence the effectiveness and responsiveness of metformin in cancer therapy. For example, few studies on metformin transporters, OCT, PMAT and MATE have provided insight into the accumulation of metformin within cancer cells to determine the specificity for cancer cells and whether normal non-cancerous cells are immune to these effects of metformin. The tissue expression of transporters mediating metformin uptake varies between normal and tumor cells and may be affected by various drugs. Poor uptake of metformin in target cells may limit its potential to treat cancer [90]. The heterogeneity of transporter expression in different cancers and how to obtain safe and effective doses of metformin in the treatment of cancer are areas of interest [332]. As with other chemotherapeutic treatment modalities, the long-term administration of metformin may be challenged by treatment-related drug resistance. Reports show that MCF7 breast cancer cells become cross-resistant to metformin and tamoxifen after long-term metformin therapy and are dependent on constitutive activation of Akt-Snail1-E-cadherin signaling [333]. Existing studies have shown inconsistent marker expression and survival outcomes for metformin as an anti-cancer agent in different settings. Variations in study design and potential biases, such as time affected by prior treatment and other relevant confounding factors, contributed to the variability of results [334].

Multiple factors influence the sensitivity of cancer cells to metformin, such as genetics, microenvironment, metabolic environment, biodistribution and tissue specificity [170]. It was demonstrated that the culture medium altered the sensitivity of cancer cells to metformin, as cells cultured in DMEM required up to 10 mM metformin to inhibit proliferation, whereas tumor cells cultured in an RPMI medium required a lower dose of metformin. At low glucose concentrations in cells lacking AMPK/LKB1, metformin is more sensitive to growth inhibition. Dietary restriction with intermittent fasting has been shown to enhance the response to metformin and metformin appears to impair tumor growth only when administered during fasting-induced hypoglycemia [335]. These are all issues that should be considered when treating cancer with metformin.

## 6. Conclusions

Metformin appears to be a new hope for HM treatment, and metformin acts as a pleiotropic compound that works together to prevent tumorigenesis via various pathways. The anti-cancer mechanism introduces the modulatory effects of metformin on multiple signaling pathways, including the activation of AMPK-dependent and AMPK-independent pathways, the improvement of cancer cell microenvironment, the amelioration of inflammatory responses and a reduction in tumor vascular metastasis. In addition, combining current anti-cancer drugs with metformin may improve their efficacy and reduce adverse drug reactions. Therefore, metformin can also be further investigated as an adjuvant therapeutic agent for HM. The field of oncology is among the most rigorous fields that use the gold standard of evidence in randomized controlled trials. Therefore, large, randomized, double-blind and placebo-controlled studies are necessary in order to find the best combination strategy by identifying the optimal dose of metformin, combination of drugs to avoid adverse events and potential biomarkers to implement this strategy in a precise oncological context. Ultimately, the efficacy of metformin in HM will be tested and the utility of the drug will be clarified.

## Figures and Tables

**Table 1:** Summary of preclinical (in vivo) use of metformin in hematological malignancies models.

Research Subjects	Models	Metformin Dosage	Year	Joint Effect of Other Drugs	Effects	Ref.
NCG mice	Acute Myelocytic Leukemia	40 mg/kg/day i.v., 4 days	2022	Ara-C	Metformin potentiated the anti-tumor efficacy of Ara-C in vivo in an NCG immunodeficient mouse xenograft model by inhibiting the mitochondrial transfer and OXPHOS activity in the engrafted human AML cells.	[195]
Female nude mice (aged 5 weeks; average weight, 16 g)	Acute Myelocytic Leukemia	200 mg/kg i.p., 14 days	2022	Venetoclax	Metformin downregulated the expression of anti-apoptotic proteins Mcl-1 and Bcl-xl by inhibiting protein production, and shows a synergistic anti-tumor effect with ABT-199 in acute myeloid leukemia.	[196]
Female C57BL/KaLwRij mice (aged 5, 6 weeks)	Multiple Myeloma	2.5 mg/mL p.o., 4 weeks	2022	-	Metformin increased OPN expression in preosteoblasts, increasing myeloma cell adherence.	[197]
MDR canines were male/neutered with recurrent B-cell lymphoma.	Lymphoma	250 mg OD or 500 mg BID p.o., 0–184 days	2022	CHOP or Doxorubicin	Metformin reduced MDR protein markers in all canines in the study.	[198]
BALC female nude mice (SPF level)	Acute Myelocytic Leukemia	125 mg/kg i.p., 21 days	2020	Ara-C	The synergistic anti-tumor effect of Ara-C/metformin in AML was via inhibiting the mTORC1/P70S6K pathway.	[199]
SCID mice (aged 6–8 weeks)	Burkitt’s Lymphoma	2 μg/mL p.o., 3 months	2020	Rituximab	Metformin in combination with rituximab showed improved survival compared with rituximab monotherapy.	[200]
NOD/SCID mice (aged 5 weeks)	Multiple Myeloma	250 mg/kg/day p.o., 21 days	2018	-	Metformin inhibited the proliferation of myeloma cells by inducing autophagy and cell-cycle arrest. The molecular mechanism involved the dual repression of mTORC1 and mTORC2 pathways via AMPK activation.	[201]
mice	Multiple Myeloma	600 μg/mL i.v., 18 or 27 days	2015	Bortezomib	Metformin suppressed GRP78, and supported the pharmacologic repositioning of metformin to enhance the anti-myeloma benefit of bortezomib.	[194]
NOD/SCID CB17-strain 394 (white) mice (aged 5, 6 weeks)	Multiple Myeloma	125 mg/kg i.p., 1 week	2015	Ritonavir	Ritonavir and metformin effectively suppressed AKT and mTORC1 phosphorylation and pro-survival BCL-2 family member MCL-1 expression in multiple myeloma cell lines.	[202]
male CB17/SCID mice (aged 4 weeks)	Multiple Myeloma	200 mg/kg/day i.p., 21 days	2015	Dexamethasone	Metformin inhibited multiple myeloma cell proliferation via the IGF-1R/PI3K/AKT/mTOR signaling pathway.	[191]
*Pten*-deficient mice (tPTEN−/−)	Lymphoma	2 mg/mouse/day i.p., 18 days	2013	-	Metformin strongly decreased the growth of luciferase-expressing tPTEN−/− cells xenografted in nude mice.	[203]
nude mice (aged 5–6 weeks)	Lymphoma	4 mg/kg/day (i.p.) or 3 mg/kg/day (p.o.) for 21 days	2012	Doxorubicin or Temsirolimus	Metformin induced AMPK activation, mTOR inhibition and remarkably blocked tumor growth in murine lymphoma xenografts.	[193]

i.p.: intraperitoneal injection; i.v.: intravenous injection; p.o.: preoral; OD: once daily; BID: twice daily; CHOP: four-drug chemotherapy cocktail given over 19 weeks including Cyclophosphamide, Doxorubicin (Adriamycin), Vincristine, Prednisone; Ara-C: cytarabine; AMPK: adenosine monophosphate-activated protein kinase; mTOR: mammalian target of rapamycin; AKT: protein kinase B; PI3K: phosphoinositide 3-kinase; OXPHOS: oxidative phosphorylation; BCL-2: B-cell lymphoma-2; MDR: multidrug resistance; GRP78: glucose-regulated protein 78; AML: acute myelocytic leukemia; OPN: osteopontin; MCL-1: myeloid cell leukemia-1; IGF-1R: insulin-like growth factor-1 receptor; PTEN: phosphatase and tensin homolog deleted on chromosome 10; mTORC1: mechanistic target of rapamycin complex 1; P70S6K: 70 kDa ribosomal protein S6 kinase; NCG: NOD/SCID/IL-2rynull; Bcl-xl: B-cell lymphoma xl; ABT-199: venetoclax; SCID: severe compromised immunodeficiency; NOD: non-obese diabetes.

**Table 2:** Summary of preclinical (in vitro) use of metformin in leukemia model.

Research Subjects	Models	Metformin Dosage	Year	Joint Effect of Other Drugs	Effects	Ref.
U937 and HL-60 cells	AML	5 mM, 24 h	2022	Ara-C	Metformin inhibited mitochondrial transfer and significantly enhanced the chemosensitivity of AML cells co-cultured with BMSCs.	[195]
LAMA-84s, LAMA-84r and K562 cells	CML	2.5–80 mM, 48 h	2022	thymoquinone	Metformin and thymoquinone monotherapies possessed a significant anti-leukemic effect that was more pronounced when combinatorial therapies are applied.	[204]
K562, K562TRBSR, MOLM13, THP1, HEL, HL60, HL60TRBSR, OCI/AML3 cells, KG1 cells and HEK293T.	AML	5 mM, 72 h	2022	-	AML cells with an MLL/AF9 genotype have a high dependency on OXPHOS and could be therapeutically targeted by metformin.	[205]
697 cells	ALL	0–15 mM, 48 h	2022	cisplatin	Metformin and cisplatin exerted a cytotoxic effect on 697 cells. When both drugs were combined they demonstrated antagonistic effects.	[206]
KG-1, Kasumi-1 and THP-1 cells	AML	0–2 mM, 48 h	2022	venetoclax	Metformin downregulated the expression of anti-apoptotic proteins Mcl-1 and Bcl-xl by inhibiting protein production, and showed a synergistic anti-tumor effect with ABT-199 in acute myeloid leukemia.	[196]
NB4, KG1 and KG1A cell lines	Leukemia	10 mM, 24 h or 72 h	2021	MCL1 Inhibitor S63845	A combined treatment with metformin and S63845 had a stronger inhibitory effect on AML cell oxidative phosphorylation and glycolysis rate and, consequently, on cellular ATP levels. The apoptosis induced by treatment was related to the change of ROS level.	[207]
K562 and KU812 cells	CML	0–10 mM, 48 h	2021	nilotinib	Metformin was effective in decreasing phosphorylated JNK levels, resulting in the restoration of nilotinib sensitivity.	[208]
OCI/AML2, K562, OCI/AML3, and THP-1 cells	Leukemia	0–10 mM, 24–72 h	2020	TP-0903	Metformin inhibited phosphorylation-dependent activation of TAM RTKs, which regulate molecular pathways associated with leukemia.	[209]
HL-60 and THP-1 cells	AML	0–12 mM, 24–72 h	2020	Ara-C	The synergistic anti-tumor effect of Ara-C and metformin in AML was achieved via inhibition of the mTORC1/P70S6K pathway.	[199]
SKM-1 cells	AML-MDS	0–20 mM, 24–72 h	2019	-	Metformin inhibited proliferation of SKM-1 cells, potentially via an AMPK-mediated cell cycle arrest.	[210]
chemoresistant AML patients	AML	10 mM, 24 h	2019	Cytarabine, Venetoclax	Metformin decreased therapy-resistant-AML cell oxidative phosphorylation in vitro, while co-treatment with cytarabine and venetoclax slightly increased the effect.	[211]
K562 cells	CML	0–30 mM, 48 h	2019	-	Metformin can inhibit the growth and proliferation of K562 cells and promote the apoptosis of K562 cells by inhibiting glycolysis energy metabolism. The PI3K/Akt/mTOR signaling pathway may be one of the molecular mechanisms of metformin on k562 cells.	[212]
U937 and THP-1 cells	AML	0–10 mM, 24 h or 48 h	2018	Diflunisal, Diclofenac	Low concentrations of metformin and the two NSAIDs diclofenac and diflunisal exerted a synergistic inhibitory effect on AML proliferation and induced apoptosis, most likely by blocking tumor-cell metabolism.	[213]
HL-60 cells	APL	0–1 μM, 24 h, 48 h, and 72 h	2018	Paclitaxel	The combination of paclitaxel and metformin triggered differentiation and apoptosis according to gene expression changes.	[214]
HL60 cells	APL	15 μM, 150 μM, and 1.5 mM, 4 h	2018	-	Low concentrations (15 and 150 µM) increased both oxidative phosphorylation and the oxidative stress response, acting on the AMPK/Sirt1 pathway, while high concentration (1.5 mM) inhibited the respiratory chain and altered cell morphology, becoming toxic to the cells.	[215]
Dami and MEG-01 cells	AMKL	4 mM, 0–72 h	2017	-	Metformin inhibited the proliferation and induced the apoptosis of human megakaryoblastic cell lines.	[216]
K562 and A301 cells	CML ALL	0–25 mM, 24 h	2017	Vincristine	AMPK activation was critical to metformin’s effects on vincristine-induced apoptosis.	[217]
MV4-11, MOLM-14, OCI-AML3, Nomo-1, THP-1 and HL-60 cells	AML	10 mM, 24 h or 48 h	2016	6-Benzylthioinosine	The combination of 6-BT with metformin resulted in significant cytotoxicity (60–70%) in monocytic AML cell lines and was associated with the inhibition of FLT3-ITD activated STAT5 and reduced c-Myc and GLUT-1 expression.	[218]
OCI-AML3, REH, NALM-6-6, and KBM5 cells	AML ALL CML	0–10 mM, 0–16 h	2016	ABT-737	Inhibition of mitochondrial metabolism by metformin or phenformin was associated with increased leukemia cell susceptibility to induction of intrinsic apoptosis.	[219]
EHEB, JVM-2 and MEC-2 cells	Leukemic	0.1–10 mM, 0–48 h	2016	Sodium Dichloroacetate	The combination of metformin and DCA increased the cytotoxicity in the B-type leukemia cell line and the cell culture derived from B-CLL patients, which was the result of cell inhibition and apoptosis promotion.	[220]
MV4-11(FLT3-ITD positive) and THP-1(FLT3-ITD negative) cells	AML	0.2–16 mM, 0–72 h	2015	Sorafenib	In the presence of metformin, the anti-cancer potential of sorafenib, accompanied by increased LC3 levels, was found to be synergistically enhanced with the remarkably reduced protein expression of the mTOR/p70S6K/4EBP1 pathway, while not appreciably altering the cell cycle.	[221]
SUP-B15, K562 and K562R cells	ALL CML	0–50 mM, 0–72 h	2015	-	Metformin mediated anti-leukemia effects by proapoptosis and inhibition of mTORC1 signaling, potentiating the anti-cancer efficacy of imatinib in Ph+ ALL cells. Metformin-induced autophagy was associated with the activation of the ERK pathway.	[222]
10E1-CEM cells	ALL	10 mM, 0–72 h	2015	-	Metformin induced cell-cycle arrest and apoptosis in drug-resistant leukemia cells.	[223]
Kassumi, NB-4, THP-1, ML-2, K562, Jurkat, Raji and HUT-78 cells	AML ALL	0–8 μM, 0–48 h	2014	-	Metformin may be involved in the downregulation of FOXM1. Metformin promoted the apoptosis of ML-2 cells, induced cell-cycle arrest at the G0/G1 and G2/M phases, and inhibited proliferation.	[224]
CCRF-CEM, Jurkat, REH and NALM6 cells	ALL	0–10 mM, 48 h	2013	-	Metformin induced ALL cell death by triggering ER and proteotoxic stress and simultaneously down-regulating the physiologic UPR response responsible for effectively buffering proteotoxic stress.	[225]
Kasumi-1, SKNO-1, HL-60, KG-1a and NB4 cells	AML APL	0–5 mM, 0–72 h	2012	ATRA	The synergism between metformin and ATRA triggered the maturation pathway in APL cells.	[226]

ALL: acute lymphocytic leukemia; AML: acute myeloid leukemia; APL: acute promyelocytic leukemia; CML: chronic myeloid leukemia; CLL: chronic lymphocytic leukemia; AMKL: acute megakaryocytic leukemia; MDS: myelodysplastic syndrome; Ara-C: cytarabine; ATRA: all-trans-retinoic acid; BMSCs: bone marrow mesenchymal stem cells; OXPHOS: oxidative phosphorylation; UPR: unfolded protein response; ER: endoplasmic reticulum; FOXM1: forkhead box protein M1; 4EBP1: 4e-binding protein 1; ATP: adenosine triphosphate; ROS: reactive oxygen species; mTORC1: mammalian target of rapamycin complex 1; P70S6K: p70S6 kinase; AMPK: AMP-activated protein kinase; PI3K: phosphatidylinositol 3 kinase; AKT: protein kinase B; mTOR: mammalian target of rapamycin; NSAIDs: non-steroidal anti-inflammatory drugs; STAT5: transcription 5; ABT-199: venetoclax; MLL/AF9: mixed-lineage leukaemia-AF9; FLT3-ITD: flt3-internal tandem duplication; LC3: light chain 3; DCA: deoxycholic; GLUT-1: glucose transporter-1; Bcl-xl: B-cell lymphoma-xl; Mcl-1: myeloid cell leukemia-1; TAM RTKs: TAM receptor tyrosine kinases.

**Table 3:** Summary of preclinical (in vitro) use of metformin in lymphoma model.

Research Subjects	Models	Metformin Dosage	Year	Joint Effect of Other Drugs	Effects	Ref.
OCI-LY-10, DB and THP-1 cells	Lymphoma	200 μM, 72 h	2022	-	Metformin could target altered lipid metabolism and decrease M2 macrophages in DLBCL, especially in CD5^+^ non-DE lymphoma.	[227]
DAUDI cells	Burkitt‘s lymphoma	10 mM, 0–72 h	2021	-	Metformin could induce cell death in BL cells by stressing cellular metabolism via the induction of GLUT1, PKM2, and LDHA.	[228]
Raji, U2392 and RL cell lines	Lymphoma	4, 8, or 16 mM, 0–72 h	2020	Rituximab	Metformin caused cell-cycle arrest in the G1 phase. Metformin induced apoptosis, ROS production, and increased mitochondrial membrane permeability. Metformin exhibited additive/synergistic effects when combined with traditional chemotherapy or rituximab in vitro.	[200]
Hut78, H9 and HH cells	Lymphoma	1 or 10 mM, 2 h	2019	-	Metformin inhibited mTORC1 signaling and migration of SS cells induced by SDF-1.	[229]
Daudi, SUDHL-4 and Jeko-1 cells	Lymphoma	0–10 mM, 48 h or 7 days	2018	Venetoclax, and BAY-1143572	Metformin inhibited oxidative phosphorylation in lymphoma cells. Metformin increased caspase 3/7 activity in venetoclax and BAY-1143572-treated lymphoma cells. Metformin showed potentiation with venetoclax and BAY-1143572 in a cell-type-dependent manner.	[230]
BC3 and BCBL1 cells	Primary exudative lymphoma	15, 20 and 30 mM, 24 h	2017	Bortezomib	The cytotoxic effect of metformin was correlated with intracellular reactive oxygen species reduction, activation of AMPK and the inhibition of pro-survival pathways such as mTOR and STAT3. Metformin altered UPR activated by bortezomib, leading to a reduced expression of BiP, upregulation of CHOP and downregulation of Bcl-2.	[231]
Jurkat, Sil-ALL, SupT1 and Ke37 cells	Lymphoma	0–10 mM, 0–48 h	2013	2-deoxyglucose	Metformin synergized with 2DG to impair tumour cell survival.	[203]
SU-DHL-4, Nalmawa, DB, SU-DHL-5, Daudi, Jurkat, 6T-CEM, Kappas, H9 and HUT78 cells	Lymphoma	0–40 mM, 0–72 h	2012	-	Metformin-induced AMPK activation was associated with the inhibition of mTOR signaling without involving AKT.	[193]

2DG: 2-deoxyglucose; AKT: protein kinase B; mTOR: mammalian target of rapamycin; AMPK: AMP-activated protein kinase; STAT3: transcription 3; UPR: unfolded protein response; DLBCL: diffuse large B-cell lymphoma; mTORC1: mammalian target of rapamycin complex 1; Bcl-2: B-cell lymphoma-2; DE: MYC/BCL2 double expressor; GLUT1: glucose transporter 1; PKM2: Pyruvate kinase M2; ROS: reactive oxygen species; SDF-1: stromal cell-derived factor-1; LDHA: Lactate Dehydrogenase A; CHOP: C/EBP homologous protein.

**Table 4:** Summary of preclinical (in vitro) use of metformin in myeloma model.

Research Subjects	Models	Metformin Dosage	Year	Joint Effect of Other Drugs	Effects	Ref.
Jeko-1 cells	MCL	10 mM, 0–72 h	2022	Bortezomib	Metformin treatment induced the resistance of cancer cells to the proteasome inhibitor Bortezomib by impairing the activity and assembly of the 26S proteasome complexes.	[232]
5TGM1, MM.1S cells	MM	0–10 mM, 48 h	2022	-	Metformin increased OPN expression in preosteoblasts, increasing myeloma cell adherence.	[197]
RPMI-8226 cells	MM	0–40 mM, 48 h	2020	Melphalan	Metformin could promote DNA damage induced by melphalan and decreased the concentration of ATP in the process of repairing DNA damage to hinder the anti-apoptotic process of tumor cells.	[233]
L363 and RPMI-8226 cells	MM	0–10 mM, 0–72 h	2020	-	Metformin inhibited HIF-1 signaling in MM cells, and the effect of metformin was mainly oxygen dependent. Metformin triggered the growth arrest without inducing apoptosis in either normoxic or hypoxic conditions.	[234]
I-8266, U266- B1, MM.1S, OPM1, OPM2, ANBL6, OCI-MY5, JJN3, KP6, DP6, KAS-6/1, KMS12PE, K562 and NALM6 cells	MM CML ALL	0–80 mM, 0–72 h	2019	-	Metformin specifically decreased IL-6R expression which is mediated via AMPK, mTOR, and miR34a.	[92]
RPMI8226, ARP-1 and OPM2 cells	MM	0–40 mM, 24 h	2019	PFK15	Metformin was found to inhibit PFKFB3 protein expression. PFK15 also demonstrated a synergistic effect with metformin to eliminate MM cells.	[235]
U266 cells	MM	5–50 mM, 0–72 h	2018	-	Metformin could inhibit cell proliferation and induced U266 cell apoptosis via the mitochondrial apoptotic pathway.	[236]
RPMI-8226 and U266 cells	MM	0–80 mM, 0–72 h	2018	-	Metformin inhibited the proliferation of myeloma cells by inducing autophagy and cell-cycle arrest. The molecular mechanism involved the dual repression of mTORC1 and mTORC2 pathways via AMPK activation.	[201]
U266, RPMI8226, LP-1 and NCI-H929 cells	MM	20 mM, 24 h	2018	FTY720	Exposure to metformin in combination with FTY720 potently induced apoptosis in MM cells in a ROS-dependent manner.	[237]
RPMI-8226 and U266 cells	MM	0–80 mM, 0–72 h	2017	-	Metformin can inhibit the proliferation and induce apoptosis of RPMI8226 and U266 cell lines, which may be related to downregulation of the STAT3 signal transduction pathway.	[238]
RPMI-8226 cells	MM	500 μM, 24 h	2015	Bortezomib	Metformin inhibited GRP78 to enhance the anti-myeloma effect of bortezomib.	[194]
KMS11, L363, and JJN3 cells	MM	0–5 mM, 0–72 h	2015	Ritonavir	Ritonavir and metformin effectively suppressed AKT and mTORC1 phosphorylation and prosurvival BCL-2 family member MCL-1 expression in multiple myeloma cell lines.	[202]
RPMI8226, MM.1S, MM.1R, and U266 cells	MM	0–80 mM, 0–72 h	2015	Dexamethasone	Metformin inhibitedMM cell proliferation via the IGF-1R/PI3K/AKT/mTOR signaling pathway.	[191]

MCL: myeloid cell leukemia; MM: multiple myeloma; CML: chronic myeloid leukemia; ALL: acute lymphocytic leukemia; PI3K: phosphatidylinositol 3 kinase; AKT: protein kinase B; PFK15: (1-(Pyridin-4-yl)-3-(quinolin-2-yl)prop-2-en-1-one); IGF-1R: insulin-like growth factor-1 receptor; Mcl-1: myeloid cell leukemia-1; mTOR: mammalian target of rapamycin; mTORC1: mammalian target of rapamycin complex 1; ATP: adenosine triphosphate; AMPK: AMP-activated protein kinase; STAT3: transcription 3; BCL-2: B-cell lymphoma-2; GRP78: glucose-regulated protein 78; FTY720: fingolimod; OPN: osteopontin; DNA: deoxyribonucleic acid; HIF-1: hypoxia-inducible factor-1; IL-6R: IL-6 receptor; PFKFB3: 6-phosphofructo-2-kinase/fructose-2,6-biphosphatase; ROS: reactive oxygen species.

**Table 5:** Metformin-based combinatorial therapy in the treatment of HM.

NCT Number	Official Title	Actual Enrollment	Status	Type of Disease	Combination Treatment	Phase
03118128	Effect of the Addition of Metformin Hydrochloride on the Prognosis of Patients With B-cell Precursor (Ph+ Negative) Acute Lymphoblastic Leukemia with High Expression of ABCB1 Gene	102	Completed	ALL	Conventional Chemotherapy	NA
01750567	A Phase II Pilot Study of Metformin Therapy in Patients with Relapsed Chronic Lymphocytic Leukemia and Un-treated CLL Patients with Genomic Deletion 11q	40	Recruiting	Relapsed CLL	None	Phase 2
05326984	Effect of Metformin on ABCB1 and AMPK Expression in Adolescents with Newly Diagnosed Acute Lymphoblastic Leukemia	20	Recruiting	ALL	Conventional Chemotherapy	NA
04741945	STOP-LEUKEMIA: Repurposing Metformin as a Leukemia-preventive Drug in CCUS and LR-MDS	24	Recruiting	Preleukemia, MDS, Cytopenia	None	Phase 2
01324180	A Phase I Window, Dose Escalating and Safety Trial of Metformin in Combination with Induction Chemotherapy in Relapsed Refractory Acute Lymphoblastic Leukemia: Metformin with Induction Chemotherapy of Vincristine, Dexamethasone, Doxorubicin, and PEG-asparaginase (VPLD)	14	Completed	ALL	VLPD	Phase 1
02948283	A Pilot Feasibility Study of Metformin/Ritonavir Combination Treatment in Patients with Relapsed/Refractory Multiple Myeloma or Chronic Lymphocytic Leukemia	3	Completed	R/R MM, R/R CLL	Ritonavir	Phase 1
01486043	Metformin as an Adjunctive Therapy for Transient Hyperglycemia in Patients with Acute Lymphoblastic Leukemia During Induction Chemotherapy	4	Terminated	ALL	Insulin	NA
01849276	A Phase I Study of Metformin and Cytarabine for the Treatment of Relapsed/Refractory Acute Myeloid Leukemia	2	Terminated	R/R AML	Cytarabine	Phase 1
00659568	A Phase I Study of Temsirolimus in Combination with Metformin in Advanced Solid Tumours	28	Completed	Lymphoma	Temsirolimus	Phase 1
02967276	Phase II Trial, Open Label, Clinical Activity of Metformin in Combination with High-dose of Dexamethasone (HDdexa) in Patients with Relapsed/Refractory Multiple Myeloma	28	Unknown	MM	High doses of dexamethasone	Phase 2
04850846	A Randomized Placebo-Controlled Phase 2 Study of Metformin for the Prevention of Progression of Monoclonal Gammopathy of Undetermined Significance and Smoldering Multiple Myeloma	80	Recruiting	MGUS, SMM	None	Phase 2
03829020	An Open-Label Phase 1 Study of Metformin and Nelfinavir in Combination with Bortezomib in Patients with Relapsed and/or Refractory Multiple Myeloma	9	Active, not recruiting	R/R PCM	Bortezomib, Nelfinavir Mesylate	Phase 1
03200015	Effect of Metformin in Combination with R-CHOP for the First Line Treatment of Patients with Diffuse Large B-cell Lymphoma	15	Unknown	DLBCL	RCHOP	Phase 2
02815397	A Phase II Study Evaluating the Efficacy and Safety of Metformin in Combination with Standard Induction Therapy (DA-EPOCH-R) for Previously Untreated C-myc+ Diffuse Large B-Cell Lymphoma	2	Terminated	DLBCL	DA-EPOCHR	Phase 2
03600363	A Prospective Randomized Controlled Phase II Clinical Trial of Metformin in the Maintenance Therapy of High Risk Diffuse Large B Lymphoma/Stage III Follicular Lymphoma Patients with Complete Remission	250	Unknown	DLBCL, Stage III FL	RCHOP	Phase 2
02531308	A Phase ll Study Evaluating the Efficacy and Safety of Metformin in Combination with Standard Induction Therapy (RM-CHOP) for Previously Untreated Aggressive Diffuse Large B-cell Lymphoma	5	Terminated	DLBCL	RCHOP	Phase 2
02145559	A Pharmacodynamic Study of Sirolimus and Metformin in Patients with Advanced Solid Tumors	24	Completed	Lymphoma	Sirolimus	Phase 1

NA: Not Applicable; CCUS: clonal cytopenia of undetermined significance; MDS: myelodysplastic syndrome; SMM: smoldering multiple myeloma; Refractory PCM: refractory plasma cell myeloma; RCHOP: rituximab, cyclophosphamide, doxorubicin, vincristine and prednisone; DA-EPOCHR: dose-adjusted etoposide, prednisone, vincristine, cyclophosphamide, doxorubicin, rituximab; Stage III FL: Stage III follicular Lymphoma; VPLD: vincristine, dexamethasone, doxorubicin and PEG asparaginase; Conventional Chemotherapy: the remission induction chemotherapy included a steroid pre-phase of 7 days of prednisone 60 mg/m^2^. The proper remission induction phase consisted of prednisone 60 mg/m^2^ daily from day 0 to 28; Vincristine 1.5 mg/m^2^ on days 0, 7, 14 and 21; Doxorubicin 25 mg/m^2^ on days 0, 7, 21; L-asparaginase 10,000 U/m^2^ on days 2, 4, 6, 8, 10 and 12. Etoposide 300 mg/m^2^ and cytarabine 300 mg/m^2^ on days 22, 25 and 29. Intrathecal chemotherapy was administered on days 0, 7, 14 and 21.

## Data Availability

Not applicable.
